# Plastome-Wide Rearrangements and Gene Losses in Carnivorous Droseraceae

**DOI:** 10.1093/gbe/evz005

**Published:** 2019-01-10

**Authors:** Paul G Nevill, Katharine A Howell, Adam T Cross, Anna V Williams, Xiao Zhong, Julian Tonti-Filippini, Laura M Boykin, Kingsley W Dixon, Ian Small

**Affiliations:** 1 ARC Centre for Mine Site Restoration, School of Molecular and Life Sciences, Curtin University, Bentley, Western Australia, Australia; 2 School of Plant Biology, The University of Western Australia, Crawley, Western Australia, Australia; 3 Kings Park and Botanic Garden, Kings Park, Western Australia, Australia; 4 Australian Research Council Centre of Excellence in Plant Energy Biology, The University of Western Australia, Crawley, Western Australia, Australia; 5 School of Molecular Sciences, The University of Western Australia, Crawley, Western Australia, Australia; 6 The University of Notre Dame, Fremantle, Western Australia, Australia

**Keywords:** carnivorous plants, chloroplast genome, Droseraceae, gene loss, intron loss

## Abstract

The plastid genomes of four related carnivorous plants (*Drosera regia*, *Drosera erythrorhiza*, *Aldrovanda vesiculosa*, and *Dionaea muscipula*) were sequenced to examine changes potentially induced by the transition to carnivory. The plastid genomes of the Droseraceae show multiple rearrangements, gene losses, and large expansions or contractions of the inverted repeat. All the *ndh* genes are lost or nonfunctional, as well as in some of the species, *clpP1*, *ycf1*, *ycf2* and some tRNA genes. Uniquely, among land plants, the *trnK* gene has no intron. Carnivory in the Droseraceae coincides with changes in plastid gene content similar to those induced by parasitism and mycoheterotrophy, suggesting parallel changes in chloroplast function due to the similar switch from autotrophy to (mixo-) heterotrophy. A molecular phylogeny of the taxa based on all shared plastid genes indicates that the “snap-traps” of *Aldrovanda* and *Dionaea* have a common origin.

## Introduction

Carnivorous plants derive a range of amino acids, peptides, macro-, and micro-nutrients from captured prey, and studies on numerous species indicate that significant resources are invested in the production of trapping organs (Plummer and Kethley 1964; Dixon et al. 1980; Adamec 1997; Adlassnig et al. 2012; Adamec 2013; Fasbender et al. 2017). Carnivory is most successful in open habitats on nutrient-poor soils and in oligotrophic aquatic environments, where it is likely to provide a competitive advantage (Givnish et al. 1984; Pavlovič and Saganová 2015). It has arisen independently at least nine times in five different orders (Caryophyllales, Ericales, Lamiales, Oxalidales, and Poales) (Albert et al. 1992; Givnish 2015), and around 700 species are currently considered carnivorous (Ellison and Adamec 2017).

Droseraceae is the largest family of carnivorous plants, comprising three extant genera with distinct morphologies: *Drosera*, *Dionaea*, and *Aldrovanda*. Although *Aldrovanda* and *Dionaea* are both monotypic, *Drosera* rivals *Utricularia* of the Lentibulariaceae as the largest carnivorous genus, with around 250 members (Gonella et al. 2016; Fleischmann et al. 2017). Members of the cosmopolitan *Drosera* vary extensively in their specific morphology, but all have highly modified leaves lined with tentaclelike glandular trichomes that secrete a sticky substance to ensnare and digest prey. Tentacle-bending and enzyme secretion are triggered by prey movements and chemical cues that induce action potentials in the contacting tentacles and subsequently local accumulation of jasmonates (Krausko et al. 2017). *Dionaea* and *Aldrovanda* also possess highly modified leaves, but unlike *Drosera* have snap-traps that close rapidly upon excitation (Poppinga et al. 2013). *Dionaea muscipula* is terrestrial and is restricted to a small area of the southeastern United States (Bailey and McPherson 2014), whereas *Aldrovandavesiculosa* is a submerged free-floating aquatic found in Europe, Asia, Australia, and Africa (Cross 2012).

At the molecular level, studies of genome evolution in carnivorous lineages have found dramatic differences when compared with solely autotrophic plant species (Fukushima et al. 2017). For example, carnivorous Lentibulariaceae exhibit the smallest angiosperm nuclear genomes (Ibarra-Laclette et al. 2013; Fleischmann et al. 2014) and high nucleotide substitution rates in all genetic compartments (Jobson and Albert 2002). The plastid genome is also reduced in size, mostly due to the independent loss of genes for the plastid NAD(P)H dehydrogenase (Wicke et al. 2014). There are also altered proportions of plastid repeat DNA; a significant plastome-wide increase of substitution rates/microstructural changes; a disproportionate elevation of nonsynonymous substitutions, particularly in protein-coding genes; and shifts in selective regimes relative to noncarnivores (Wicke et al. 2014). This genome instability contrasts with the general observation that plastid genomes are highly conserved in sequence, gene content, and gene order in flowering plants (Green 2011; Wicke et al. 2011). Unusual modes of plastid genome and plastid gene evolution are rare in autotrophic lineages (Palmer et al. 1988; Cosner et al. 2004; Sloan et al. 2012). Plastome instability and gene loss appears to be associated with the switch from an autotrophy to (mixo-) heterotrophy. Elevated plastome-wide substitution rates and the loss of *ndh* genes observed in Lentibulariaceae were also found in obligate photosynthetic, parasitic plants including Orobanchaceae (Wicke et al. 2013; Frailey et al. 2018) and *Cuscuta* (McNeal, Arumugunathan, et al. 2007; McNeal, Kuehl, et al. 2007) and in mycoheterotrophs (Graham et al. 2017). This raises a question: Is the rapid evolution of plastid genomes promoted by different nutrient acquisition strategies?

Advances in high-throughput sequencing have greatly increased the ability to characterize whole genomes, particularly from organelles (Tonti-Filippini et al. 2017). Combined with analytical tools and databases that facilitate genome annotation and comparisons with previously sequenced plastid genomes, studies of genome evolution in nonmodel organisms are increasingly accessible. To explore the effect of the carnivorous syndrome on the evolution of the chloroplast, we sequenced the chloroplast genomes of the three extant genera (*Drosera*, *Dionaea*, and *Aldrovanda)* of the carnivorous plant family Droseraceae. The results of this study improve our understanding of the genomic effects of a carnivorous strategy and provide further evidence of the congruence between carnivorous and parasitic plastid genomes.

## Materials and Methods

### Plant Material and DNA Extraction

Plant material of *A. vesiculosa* L. was collected from Cape le Grande, Esperance, Western Australia (voucher: PERTH 08722560) (Cross 2012). Plant material of *Drosera erythrorhiza* Lindl. was collected from Alison Baird Reserve, Kenwick, Western Australia (voucher: PERTH 06332374). Plant material of *D.muscipula* Sol. ex J.Ellis and *Drosera regia* Stephens were collected from cultivated individuals maintained in the living collection at Kings Park and Botanic Gardens, Perth, Western Australia. Total genomic DNA was extracted from fresh material of all four species using a cetyl trimethylammonium bromide method (Doyle and Doyle 1987). Cetyl trimethylammonium bromide extraction buffer (1 ml) was added to each sample in a 2-ml tube containing a scoop of sterilized silica sand and two small glass beads. Tissue was macerated twice for 30 s at speed four (6.5 m/s) in a Thermo Savant FASTPREP FP120. All samples were treated with RNase (Qiagen), and DNA quality and quantity were assessed using a NanoDrop spectrophotometer (ND-1000; Thermo Fisher Scientific, USA), a Qubit 2.0 fluorometer (Invitrogen, Life Technologies) and via agarose gel electrophoresis. DNA samples were purified and concentrated using a DNA Clean & Concentrator-5 kit (Zymo Research).

### Illumina Sequencing

Total genomic DNA (30–150 ng) was fragmented with a Covaris S2 to a mean fragment size of 550 bp. DNA libraries were prepared using the TruSeq DNA Nano Library Prep kit (Illumina), according to the manufacturer’s instructions. Libraries were quantified by quantitative polymerase chain reaction, using a KAPA Illumina Library Quantification kit (KAPA Biosystems) and LightCycler 480 (Roche), and then pooled in approximately equimolar amounts. The pooled libraries were sequenced (2× 150 bp) using the Illumina HiSeq 1500 in rapid run mode using a TruSeq Rapid PE Cluster kit, for on-board cluster generation, and TruSeq Rapid SBS kits.

### Sequence Assembly and Annotation

For each specimen, overlapping paired-end reads were merged using the software FLASH v. 1.2.7 (Magoč and Salzberg 2011) to maximize the effective read length. Merged reads were assembled de novo using Velvet v. 1.2.08 (Zerbino and Birney 2008) with k-mer values of between 71 and 111 and the coverage cutoff optimized for each sample and k-mer value to reduce contamination by nuclear and mitochondrial reads. For *D.muscipula*, *A. vesiculosa*, and *Dr.regia*, complete contigs corresponding to the large and small single copy regions and the inverted repeat were obtained from the initial assembly. For *Dr.erythrorhiza*, five contigs were obtained (table 1). For each assembly, MUMmer v. 3.0 (Kurtz et al. 2004) was used to compare the assembled chloroplast contigs with other plastid genomes, particularly that of *Fagopyrum esculentum* (RefSeq accession NC_010776) to order the contigs correctly. The assemblies were refined by mapping the original reads to the draft genomes with bowtie2 (using default parameters) (Langmead and Salzberg 2012), verifying the assembly with pilon (Walker et al. 2014) and manual correction of any problems through the visual interface of Geneious 9.1.5 (Kearse et al. 2012). Typically, the few discrepancies found involved varying lengths of homopolymer runs or copy number of short tandem repeats. The mapping, verification, correction process was iterated as necessary. Assemblies were also attempted using two seed-extension assemblers, The Organelle Assembler v. b′2.2′ (https://git.metabarcoding.org/org-asm; last accessed February 3, 2019, parameters “–minread 5 –smallbranches 15 –seeds protChloroArabidopsis”) and NOVOPlasty v. 2.5.9, (using k-mer length 39 and the *Zea mays rbcL* gene as a seed) (Dierckxsens et al. 2017). The resulting single-contig assemblies were permuted to match canonical plastid genome arrangements by flipping the single copy regions if necessary. The genomes were initially annotated by the “Transfer Annotations” function within Geneious 9.1.5 (Kearse et al. 2012) (using *F. esculentum* as a reference at a 90% identity threshold) followed by manual curation using similarity to each other and to other previously annotated genomes as a guide. Genes were only annotated as present when there was a reasonable expectation that the gene product might be functional, that is, degenerate sequences missing conserved domains, or containing frameshifts or internal stop codons were not annotated. The full sets of unassembled reads are available as SRA accessions SRR7072322–SRR7072325 and SRR7072766–SRR7072769. The assembled, annotated sequences are available as GenBank accessions KY651214 (*Dr.erythrorhiza*), KY679199 (*Dr.regia*), KY679200 (*A. vesiculosa*), and KY679201 (*D.muscipula*).

**Table 1:** Various Statistics Concerning the Droseraceae Genome Assemblies

	Genome Size (bp)	Velvet	Organelle Assembler	NOVOPlasty	IR Length (bp)	Mapped Reads	Mean Coverage
*Dionaea muscipula*	117,589	57,689	117,692	91,604	2,798	1,313,104	1,408×
		54,555		26,001			
		*2,668					
*Aldrovanda vesiculosa*	141,568	75,654	141,583	141,573	27,040	484,787	517×
		*26,910					
		12,083					
*Drosera regia*	136,810	79,603	136,810	136,810	23,586	827,359	992×
		*23,446					
		10,364					
*Drosera erythrorhiza*	134,391	51,587	129,708	54,762	37,186	425,913	463×
		*19,096		45,674			
		*12,777		33,455			
		*5,183					
		3,954					
		3,871					

Note.—* indicates contigs that were duplicated in the final assembly as part of the inverted repeats.

### Verification of Gene Losses

Reads from samples with apparently missing plastid genes were mapped to related genomes containing the gene to verify if reads matching the gene sequence were present in the sample, but not assembled into the final genome. This was carried out using bowtie2 (Langmead and Salzberg 2012) (default parameters) and bwa v. 0.7.15 (bwa-mem algorithm, -T 25) (Li and Durbin 2009). Crossmappings of this type were done for *Dr.erythrorhiza* against *Drosera rotundifolia* and *Dr.regia*, and for all of the Droseraceae sequenced here against the *F. esculentum* genome. In no cases were additional plastid sequences found that had not been included in the assemblies. Contig pools generated during the assemblies were also checked by TBlastN (with an *e*-value cutoff of 10^−5^) using the protein sequences of ClpP1, Ycf1, Ycf2, Rpl32, and NdhA as queries. No contigs not already included in the assemblies were discovered.

### Phylogenetics

Fifty-eight proteins are encoded in each of the five Droseraceae plastid genomes and that of *F. esculentum.* Each set of six protein-coding (i.e., nucleotide) sequences was aligned independently using TranslatorX v. 1.1 (Abascal et al. 2010) to align codons with MAFFT v. 7.407 (Katoh and Standley 2013) as the alignment engine. The alignments were then trimmed with trimAl v1.4 (-automated1) (Capella-Gutierrez et al. 2009) and concatenated. Bayesian analyses were conducted using MrBayes v. pre-3.2.7 (Ronquist et al. 2012). The aln.model file consisted of 58 partitions corresponding to each protein-coding sequence and the parameters were estimated based on these partitions. The alignment and the resulting MrBayes phylogeny are available from TreeBASE (study TB2: S23621). Thirty independent analyses were run simultaneously for 30 million generations with sampling every 300 generations. Each analysis consisted of two independent runs, each utilizing four coupled Markov chains. The run convergence was monitored by finding the plateau in the likelihood scores (standard deviation of split frequencies <0.0015). The first 25% of topologies were discarded as burn-in for the estimation of a majority rule consensus topology and posterior probability for each node on the phylogenetic tree. Finally, Tracer v. 1.7.1 (Rambaut et al. 2018) was used for analyzing the trace files generated by the Bayesian MCMC runs to identify potential convergence problems. The same alignments were used to generate maximum-likelihood trees with RAxML v. 8.2.12 (Stamatakis 2014), using -m GTRGAMMAI and 1,000 rapid bootstrap inferences, and with IQ-TREE v.1.6.8 (Nguyen et al. 2015) using automatic selection of the best-fit model (Kalyaanamoorthy et al. 2017), with -alrt 100000 -bb 100000.

## Results

### Structure of the Plastid Genomes in Droseraceae

**Fig. 1.**
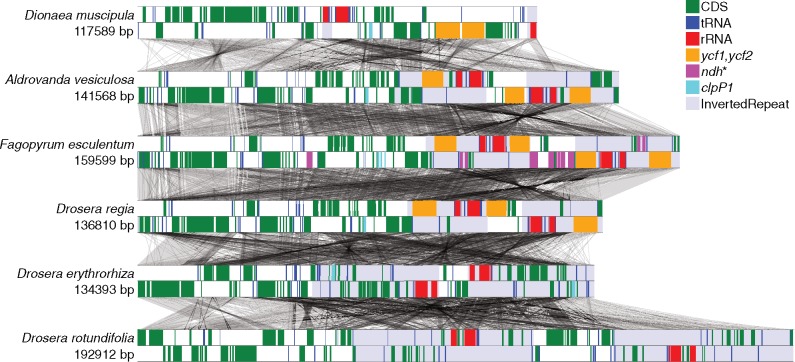
—Physical maps of the plastid genomes of Droseraceae compared with that of *Fagopyrum esculentum.* The genomes are drawn to scale. Different gene classes and the inverted repeats are indicated by colored bands. Gray lines connecting adjacent genomes indicate synteny; each line represents an indel-free sequence alignment of >40 bp in length and with >50% matching bases. The genomes have been arranged to display synteny conservation as much as possible, that is, the most divergent genomes have been placed at the top and bottom.

Illumina sequencing of libraries prepared from total DNA of *D.muscipula* (Venus’ fly trap), *A.vesiculosa* (waterwheel plant), *Dr.regia* (king sundew), and *Dr.erythrorhiza* (red ink sundew) produced between 5 and 64 million paired-end reads per sample with a length of 150 nt. For each specimen, ∼5% of the reads could be assembled into contigs homologous to the reference plastid genome of *F.esculentum* (Logacheva et al. 2008), the most closely related noncarnivorous plant whose plastid genome has been sequenced. Read coverage was relatively even across the genomes and exceeded 400× in all cases (table 1). Complete assemblies were obtained for all four species, ranging from 117,589 nt (*D.muscipula*) to 141,568 nt (*A. vesiculosa*) (fig. 1). Similar assemblies were obtained with three different assemblers (table 1). Minor unresolved discrepancies between assemblies were largely due to differences in the copy number of short tandem repeats in low complexity intergenic regions; these variations were present in the sequencing reads and may be present in vivo. These genomes are considerably smaller than the reported 192,912 nt size of the *Dr.rotundifolia* genome (RefSeq accession NC_029770). The differences in genome sizes are primarily due to large differences in the extent of the inverted repeats and secondarily due to differences in gene content.

### Expansion and Contraction of the Inverted Repeats

Based on the generally typical genome size and arrangement of *F. esculentum*, the ancestral plastid genome of the Droseraceae is likely to have contained a typical inverted repeat of 25–30 kb containing the *rrn* genes, several tRNA genes (including *trnI-GAU* and *trnA-UGC*), several genes encoding ribosomal subunits, *ndhB*, and most or all of the *ycf1* and *ycf2* coding sequences. All of the Droseraceae have a modified IR region compared with *F. esculentum*, with an extreme variation in size from under 3 kb in *D. muscipula* to almost 40 kb in *Dr.erythrorhiza* and *Dr.rotundifolia* (table 1 and fig. 1), with concomitant variation in gene content from only 1 gene entirely within the IR in *D.muscipula* (*rrn16*) up to 40 genes contained within the IR of *Dr.erythrorhiza*. These variations are not simply movements of the IR borders but include multiple gene losses and other rearrangements resulting in a lack of colinearity throughout the IR region between these genomes. The very unusual size and structure of the IR in *D.muscipula* was confirmed by polymerase chain reaction (supplementary fig. S1 and supplementary table S1, Supplementary Material online).

### Gene Content of the Plastid Genomes in Droseraceae

**Fig. 2.**
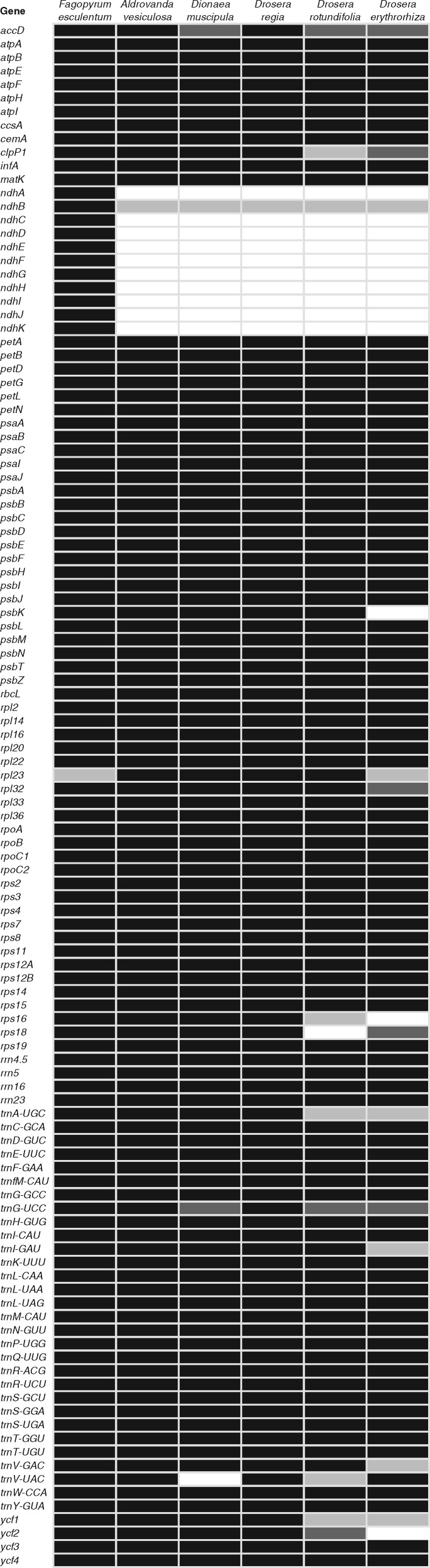
—Gene content in Droseraceae plastid genomes and in *Fagopyrum esculentum*. Genes present and apparently functional are shaded in black, doubtfully functional genes in mid-gray, almost certainly nonfunctional remnants in pale gray and missing genes in white.

**Fig. 3.**
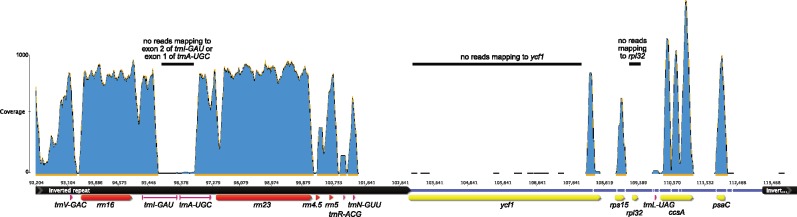
—Mapping of *Drosera erythrorhiza* reads to the *Drosera regia* genome across the inverted repeat-single copy region. Reads were mapped using bowtie2 (Langmead and Salzberg 2012) with default parameters and coverage was visualized using Geneious 9.1.5 (http://www.geneious.com, last accessed February 3, 2019, Kearse et al. 2012). Most genes in this region show extensive coverage (∼1,000×), but coverage is extremely low over the last and first exons of *trnI* and *trnA*, respectively, almost all of *ycf1*, and *rpl32*. The lack of crossmapping of raw reads to *Drosera regia* confirms that these sequences are missing from the *Drosera erythrorhiza* genome.

**Fig. 4.**
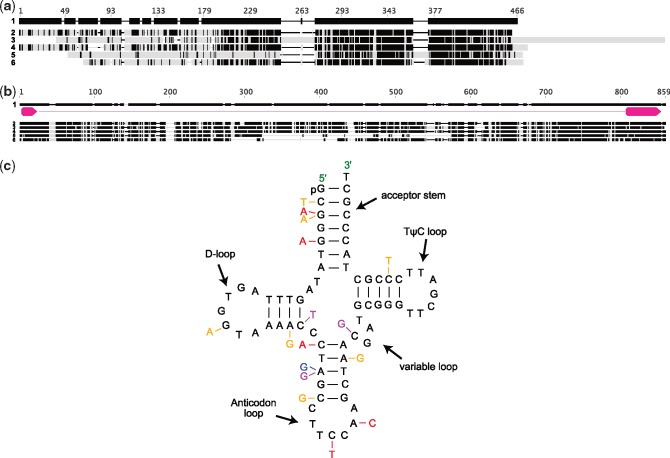
—(*a*) Alignment of the predicted *accD* protein sequences from the Droseraceae species compared with that of *Fagopyrum esculentum*, shaded to indicate conservation. Black indicates amino acids identical to those in the *F. esculentum* sequence. Sequences are numbered as follows: 1, *F. esculentum*; 2, *Aldrovanda vesiculosa*; 3, *Dionaea muscipula*; 4, *Drosera regia*; 5, *Drosera erythrorhiza*; and 6, *Drosera rotundifolia*. The sequences from *D. muscipula, Dr. erythrorhiza*, and *Dr. rotundifolia* are truncated at the N-terminus, contain large indels, and in the case of *D. muscipula*, a long C-terminal extension due to mutation of the usual stop codon*.* The functionality of these sequences is doubtful. (*b*) Alignment of the *trnG-UCC* gene from the Droseraceae species compared with that of *F. esculentum*, shaded to indicate conservation. Black indicates nucleotides identical to those in the *F. esculentum* sequence. The positions of the tRNA exons are indicated by magenta arrows. Sequences are numbered as in (*a*). Alignment and visualization was done with Geneious 9.1.5 (http://www.geneious.com, Kearse et al. 2012). (*c*) Cloverleaf representation of the *trnG-UCC* sequence from *F. esculentum*, indicating nucleotide changes in the Droseraceae (magenta for *D. muscipula*, orange for *Dr. rotundifolia*, and red for *Dr. sera erythrorhiza*). Many of these nucleotide changes would be expected to severely impact the function of the tRNA, most obviously the alteration in the anticodon in *Dr. erythrorhiza*.

The *F. esculentum* plastid genome (Logacheva et al. 2008) includes 114 unique genes, of which 11–21 are missing from the Droseraceae plastid genomes (fig. 2). The 11 plastid genes encoding subunits of the ferredoxin-plastoquinone oxidoreductase (NDH) complex are missing (or rearranged to the point that they cannot possibly be functional) from all of the Droseraceae plastid genomes. Multiple additional gene losses have occurred within the Droseraceae. Of particular interest are the loss/inactivation of the usually essential protein-encoding genes *clpP1* (supplementary fig. S2, Supplementary Material online), *ycf1*, and *ycf2* from *Dr.erythrorhiza* and *Dr.rotundifolia* and the loss of several equally essential tRNA genes: *trnA-UGC* from *Dr.erythrorhiza* and *Dr.rotundifolia*, *trnV-UAC* from *Dr.rotundifolia* and *D.muscipula*, and *trnI-GAU* from *Dr.erythrorhiza*. Gene losses were verified by mapping raw reads from the relevant sample against the nearest related genome containing the gene to make sure that apparent losses were not simply assembly errors. An example is shown in figure 3. In addition to complete gene losses, other genes have accumulated mutations, indels, and rearrangements to the point where their functionality in at least some of the species is doubtful. Examples include *accD* and *trnG-UCC* (fig. 4).

### Intron Losses

**Fig. 5.**
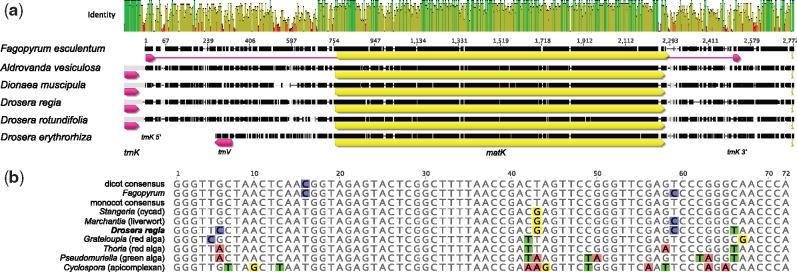
—(*a*) Alignment of the *trnK-matK-psbA* region of the Droseraceae genomes in comparison to the homologous region from *Fagopyrum esculentum*. The *matK* coding sequence is indicated in yellow, tRNA genes in magenta (*trnK* in all cases except *Drosera erythrorhiza*, where the tRNA gene shown is *trnV-GAC*; *trnK* is found elsewhere in the genome). Nucleotide identity is indicated by the plot about the alignment (green indicates 100% identity, yellow indicates 30–80%, and red indicates below 30%). Alignment and visualization was done with Geneious 9.1.5 (http://www.geneious.com, Kearse et al. 2012). (*b*) Alignment of *trnK* sequences from diverse plastids. The sequences were selected as representatives from an alignment of 1654 *trnK* and tRNA-Lys sequences from GenBank. Dicot and monocot consensus sequences represent hundreds of identical sequences from these clades. The other sequences shown are from specific accessions: *Fagopyrum esculentum* (NC_010776), *Stangeria eriopus* (JX416858.1), *Marchantia polymorpha* (M20959.1), *Drosera regia* (this work), *Grateloupia taiwanensis* (KC894740.1), *Thorea hispida* (KX284714.1), *Pseudomuriella schumacherensis* (KT199256.1), and *Cyclospora cayetanensis* (KX273388.1). The *trnK* genes in land plants (except the Droseraceae) are split by an intron between positions 37–38, so the sequences shown are the exon fusions. Those in algae (except charophytes) are not split.

In addition to losses of entire genes, there are also losses of specific introns. Both introns are missing from the *clpP1* genes of all the Droseraceae. These introns are also missing from several other plant lineages, including grasses, pines, and some dicot genera (Erixon and Oxelman 2008; Jansen et al. 2008). The intron of *rpl2* has also been lost independently from several groups of flowering plants and was reported to be missing from *Drosera filiformis* based on filter hybridization (Downie et al. 1991). We confirm the loss of this intron from all the Droseraceae. A fourth intron missing from all of the plastid genomes we have sequenced is the second (*cis*-spliced) intron of *rps12*. Again, this is not unique among flowering plants as the same intron has been lost from legumes (Jansen et al. 2008) and from parasitic plants such as *Cuscuta* (McNeal et al. 2009). Splicing of *rpl2* and *rps12* intron 2 is thought to require the intron maturase MatK (Zoschke et al. 2010), so it is interesting to note that the arrangement of the *trnK* and *matK* genes in the Droseraceae is unique. In all other land plant genomes where it is present, the *trnK* gene contains a long intron in which lies the *matK* gene. In all the Droseraceae plastid genomes, the *trnK* gene lacks an intron, and in all but *Dr.erythrorhiza*, lies near to the *matK* gene, adjacent to the sequence corresponding to the 5′ *trnK* exon in other plant plastid genomes (fig. 5*a*). The sequence of the Droseraceae *trnK* differs from that in *F. esculentum* and other flowering plants (fig. 5*b*).

### Changes in Gene Order

There are many differences in gene order and orientation between the different plastid genomes of the Droseraceae (fig. 1). Some of these rearrangements have affected the 18 cotranscribed gene sets whose order is otherwise highly conserved across flowering plants (Sugita and Sugiura 1996) (supplementary fig. S3, Supplementary Material online). A summary is shown in table 2. Some of the alterations to transcription units are due to loss of one or more of the genes normally contained within, for example, loss of the *ndh* genes disrupts three commonly conserved transcription units. Others are due to rearrangements that break the linkage between adjacent genes but without the loss of any genes, for example, the separation of the *rps11*-*rpoA* gene pair from the *rpl23*…*rpl36* gene cluster in *Aldrovanda*. As these rearrangements are unlikely to be reversible, they may be phylogenetically informative where events are shared between taxa. In this context, it is notable that *Aldrovanda* and *Dionaea* share a broken linkage between *rpl33* and *rps18*, while *Dr.erythrorhiza* and *Dr.rotundifolia* share broken linkages in the *psbB*…*petD* and *psbK*…*trnG* transcription units (specifically between *psbH*-*petB* and *psbI-trnG*, respectively).

**Table 2:** Loss or Breakage of Plastid Cotranscription Units Across the Droseraceae

Transcription Unit	*Fagopyrum*	*Aldrovanda*	*Dionaea*	*Drosera regia*	*Drosera erythrorhiza*	*Drosera rotundifolia*
*atpB-atpE*	✓	✓	✓	✓	✓	✓
*rps12-rps7*	✓	✓	✓	✓	✓	✓
*psbE-psbF-psbL-psbJ*	✓	✓	✓	✓	✓	✓
*psbD-psbC-psbZ*	✓	✓	✓	✓	✓	✓
*ndhC-ndhK-ndhJ*	✓	All *ndh* genes lost	All *ndh* genes lost	All *ndh* genes lost	All *ndh* genes lost	All *ndh* genes lost
*rrn16-trnI-trnA-rrn23-rrn4.5-rrn5*	✓	✓	One full copy only	✓	*trnI*, *trnA* partially deleted	*trnA* partially deleted
*rpoB-rpoC1-rpoC2*	✓	✓	✓	✓	✓	Broken between *rpoC1* and *rpoC2*
*rpl23-rpl2-rps19-rpl22-rps3-rpl16-rpl14-rps8-infA-rpl36-rps11-rpoA*	✓	Broken between *rpl36* and *rps11*	✓	✓	*rpl23* lost	✓
*trnE-trnY-trnD*	✓	✓	✓	✓	✓	✓
*clpP1-rps12-rpl20*	✓	✓	✓	✓	*clpP1* nonfunctional but transcription unit intact	*clpP1* nonfunctional but transcription unit intact
*petL-petG-psaJ-rpl33-rps18*	✓	Broken between *rpl33* and *rps18*	Broken between *rpl33* and *rps18*	✓	✓	✓
*psaA-psaB-rps14*	✓	✓	✓	✓	✓	✓
*psaC-ndhD*	✓	*ndhD* lost	*ndhD* lost	*ndhD* lost	*ndhD* lost	*ndhD* lost
*psbB-psbT-psbH-petB-petD*	✓	✓	✓	✓	Broken between *psbH* and *petB*	Broken between *psbH* and *petB*
*psbK-psbl-trnG*	✓	✓	✓	✓	*psbK* lost, broken between *psbI* and *trnG*	*psbK* lost, broken between *psbI* and *trnG*
*ndhA-ndhI-ndhG-ndhE-psaC*	✓	All *ndh* genes lost	All *ndh* genes lost	All *ndh* genes lost	All *ndh* genes lost	All *ndh* genes lost
*rpl32-ccsA*	✓	✓	Broken	✓	*rpl32* lost	Broken
*rps2-atpI-atpH-atpF-atpA*	✓	✓	✓	✓	✓	✓

### Phylogenetic Analyses

**Fig. 6.**
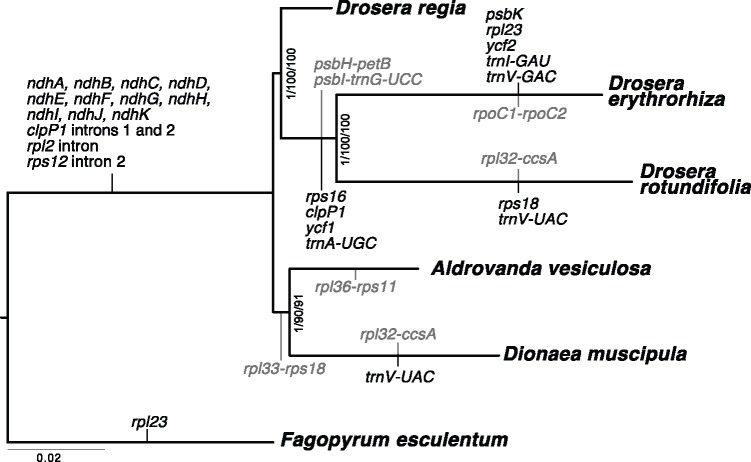
—Phylogenetic tree based on Droseraceae plastid genomes. Fifty-eight protein-coding sequences shared by all genomes in this study were aligned gene-by-gene and then the alignments were concatenated. The most probable tree topology obtained by MrBayes, RAxML, and IQ-TREE is shown with the support values (posterior probabilities or bootstrap support) shown at the nodes. The branch lengths calculated by each program were similar. Putative loss of gene function, intron losses, and breaks in cotranscription units (in gray text) are indicated against the branches where they occurred.

The coding sequences shared by the Droseraceae plastid genomes were used to reconstruct their phylogenetic relationships, using the *Fagopyrum* genome as an outgroup (fig. 6). The tree strongly supports previous phylogenetic hypotheses regarding the relationships between *Drosera*, *Dionaea*, and *Aldrovanda*, with *Aldrovanda+Dionaea* forming a sister group to *Drosera* (Cameron et al. 2002; Rivadavia et al. 2003).

## Discussion

### Confirmation That “Snap-Traps” Evolved Only Once in the Droseraceae

The Droseraceae diverged relatively early from the other noncarnivorous Caryophyllales (around 65 Ma; Heubl et al. 2006), and the age of paleobotanical finds for ancestral *Aldrovanda* species (55–38 Ma) indicate that divergence within the family also occurred relatively early (Cross 2012). There has been much discussion about the evolution of the remarkable traps of the Droseraceae and the relationships between the diverse methods for capturing live prey that are found in the three genera (Poppinga et al. 2013). In particular, it has often been questioned whether the highly distinctive active “snap-traps” found in *Dionaea* and *Aldrovanda* have a common origin or evolved independently. Phylogenetic studies utilizing nuclear markers have placed *Aldrovanda* sister to *Dionaea* within Droseraceae (bootstrap support of around 90%), supported by combined analyses of *rbcL* and nuclear 18S rDNA (Rivadavia et al. 2003), and *rbcL*, *matK*, *atpB*, and 18S rDNA (Cameron et al. 2002), as well as similarities in trichome and gland morphology (Elansary et al. 2010). Phylogenies based only on chloroplast DNA have either lacked sufficient resolution or placed *Aldrovanda* closer to members of *Drosera* (Cameron et al. 2002; Rivadavia et al. 2003), although this discrepancy has been attributed to chloroplast capture because of the incongruence between trees based on plastid genomes and nuclear sequences (Elansary et al. 2010). The molecular evidence for these relationships is now much stronger with the entire plastid genomes to analyze. Our phylogenetic analysis supported the tentative conclusions of the previous molecular and morphological studies, confirming the sister relationship between *Aldrovanda* and *Dionaea* (Elansary et al. 2010; Poppinga et al. 2013) and confirming that they form a clade that is sister to *Drosera* with the South African species *Dr.regia* sister to other *Drosera* sp. (Rivadavia et al. 2003). Our trees based on plastid sequences agree with the previous nuclear DNA phylogenies. Thus, our results do not support chloroplast capture and we suspect that the difficulty in establishing the correct phylogeny from plastid sequences in the past may be partly due to the large variations in genome structure and gene content within the Droseraceae. In particular, sequences within the inverted repeats tend to show a much reduced rate of fixation of mutations (Wolfe et al. 1987), and the extreme variation of the extent of these repeats within the Droseraceae may lead to differences in the rate of divergence of different genes in different lineages. For example, much of the *rbcL* gene, used for most of the molecular phylogenetics work to date (Cameron et al. 2002; Rivadavia et al. 2003), is within the IR in *Dr.erythrorhiza* but not in the other species studied. Similarly, we have shown that the *matK* gene, another popular choice for phylogenetic reconstructions, is in a different genomic environment in the Droseraceae than in other flowering plants, with no doubt a different set of selection pressures acting on it and its flanking sequences.

Further evidence for the relationship and origin of the Droseraceae can be found in the pattern of gene function losses. The unique loss of the *trnK* intron proves a common origin for the three genera and thus confirms the monophyly of the Droseraceae. There are no losses specific to the *Aldrovanda* + *Dionaea* clade, but the disruption of the *petL*…*rps18* transcription unit between *rpl33* and *rps18* is only seen in these two taxa and is probably a synapomorphy. The position of *Dr.regia* as sister to the rest of the *Drosera* clade is confirmed by the losses of *trnA* and *ycf1* and several other rearrangements that are shared between the northern hemisphere plant *Dr.rotundifolia* and the southern hemisphere *Dr.erythrorhiza* but not with *Dr.regia*. *Drosera regia* is restricted to a single mountain valley in South Africa and has several pleisomorphic characters (e.g., operculate pollen, a lack of stipules) that are more similar to those in *D.muscipula*, and which traditionally has resulted in it being treated as a different group to other *Drosera* (Fleischmann et al. 2017).

#### An Unusual *trnK* Gene Suggests an Unlikely Event

The original *trnK* sequence and associated intron is still present (if barely recognizable) in all the Droseraceae except *Dr.erythrorhiza*, where the 5′ exon and most of the 5′ half of the intron is missing (fig. 5). This *trnK* pseudogene is clearly not functional as it is not conserved within the Droseraceae. Instead, there has been an insertion of a new *trnK* gene, which does not contain an intron, and which is conserved within the Droseraceae and thus presumably functional. In all the Droseraceae besides *Dr.erythrorhiza*, this new *trnK* gene is adjacent to the remains of the previous one, and this is presumably the original site of the insertion. In *Dr.erythrorhiza*, the new *trnK* gene is tens of kilobases away near the *trnD* gene. The loss of the *trnK* intron, without concomitant loss of the *trnK* gene, is unique (as far as we are aware) within the terrestrial flora. Insertion of the *matK*-containing intron within *trnK* predates the colonization of the land by plants, as this intron is found throughout the charophyte clade (Hausner et al. 2006), except in a few plants (notably most ferns and many parasitic plants) that have lost *trnK* entirely (Duffy et al. 2009; McNeal et al. 2009).

We propose two possible explanations for the origin of the novel *trnK* gene in the Droseraceae. Both of them appear unlikely, but other explanations would seem even less likely. One possibility is that a spliced *trnK* transcript was reverse transcribed and reinserted into the genome. This is thought to be the mechanism by which intron losses in other plastid genes have occurred (Downie et al. 1991). However, the sequence of the Droseraceae *trnK* differs from that in other flowering plants. This hypothesis must therefore account for the fact that within a relatively short period of time (after the divergence from *Fagopyrum* but before the divergence within the Droseraceae), this sequence has accumulated several mutations not seen in other flowering plants. This may be possible if the requirement for intron splicing places a strong selective pressure on the *trnK* exons that is relaxed once splicing is no longer necessary. Alternatively, the Droseraceae *trnK* gene may be derived from horizontal transfer from another organism. Horizontal gene transfer between plant mitochondria has been documented on multiple occasions (Bergthorsson et al. 2003, 2004), and of functional tRNA genes from plastids to mitochondria (Joyce and Gray 1989). However, as far as we are aware, horizontal gene transfer has not been observed between plastids in land plants, although it has been shown in algae (Ruck et al. 2017). However, as the *matK* intron insertion into *trnK* occurred prior to the appearance of charophyte algae, if the transferred sequence was DNA, this implies the source would have to have been a noncharophyte alga. Algal plastid *trnK* sequences are reasonably similar (in some cases >90% identical) to the Droseraceae sequence, and as the Droseraceae are often associated with wet or aquatic habitats such a transfer is perhaps not entirely outside the realms of possibilities.

The *matK* gene is retained in the Droseraceae, despite the loss of the *trnK* intron. This is similar to the case in plants where *matK* is retained despite complete loss of *trnK* (Duffy et al. 2009; McNeal et al. 2009), and adds to the evidence that the MatK maturase is essential for splicing other plastid introns (Zoschke et al. 2010). Three of the introns thought to require MatK are missing from the Droseraceae plastid genomes (*trnK*, *rpl2*, and *rps12* intron 2) but several putative MatK targets remain. There is an interesting parallel in Geraniaceae mitochondria, where loss of several *nad1* introns has left the *matR* gene (usually encoded within *nad1* intron 4) as a free-standing and presumably functional gene (Grewe et al. 2016), because it is required for splicing of introns in other genes (Sultan et al. 2016).

### Functional Implications of Genome Rearrangements and Gene Losses

The main focus of this study was to explore the effect of the carnivorous syndrome on evolution of the plastid genome in Droseraceae and examine any resultant alteration in plastid function. All three extant genera in Droseraceae have highly rearranged plastid genomes, including some rarely or never before seen events. These include the losses of several genes usually thought to be essential in plant plastids. Only four protein-coding genes are consistently retained in most plastid genomes of nonphotosynthetic parasitic plants that have lost most of their plastid genes: *accD*, *ycf1*, *ycf2*, and *clpP1* (Wolfe, Morden, and Palmer 1992; Delannoy et al. 2011; Graham et al. 2017). All four genes are essential when tested by gene knockout in tobacco (Drescher et al. 2000; Kuroda and Maliga 2003; Kode et al. 2005), and yet at least one of them is missing or appears to be nonfunctional in each of the Droseraceae plastid genomes. Notably, *Dr.rotundifolia* and *Dr.erythrorhiza* appear to have lost the function of all four. Despite this rather startling set of plastid gene losses, the Droseraceae retain the ability to make fully functional chloroplasts, and indeed are highly successful at deriving carbon and energy from autotrophy even in the absence of prey capture (Bruzzese et al. 2010; Pavlovič et al. 2014). It seems probable therefore that the *accD* (required for acetyl-CoA carboxylase) and *clpP1* (required for the Clp protease) genes are functionally replaced by nuclear counterparts, either because the plastid genes have been transferred or because nuclear homologs have gained plastid functions (exemplified by the nuclear *ACC2* gene in Brassicaceae; Parker et al. 2016). The fate of *ycf1* and *ycf2* is less clear; in other plants, loss of *ycf1* and *ycf2* is not usually associated with transfer of these genes to the nucleus, and as the function of the gene products is not fully understood, what complements their loss is not known. Ycf1 has been proposed to be a key component of the general protein import channel (Kikuchi et al. 2013). However, this has been challenged (de Vries et al. 2015; Bölter and Soll 2017), one of the reasons being that multiple independent losses of this gene have occurred in plant plastids, which seems hard to reconcile with such an essential function. The Droseraceae provide an additional example that requires explanation. The *ycf1* and *ycf2* genes, when lost, are often lost in quick succession, strongly hinting that they act in the same pathway or function, and thus that the absence of both can be complemented by the same (unknown) process. There is also some indication that loss/retention of *accD* also correlates with that of *ycf1*/*ycf2* (Delannoy et al. 2011; de Vries et al. 2015), a correlation that is strengthened by our analysis of the Droseraceae genomes. A role for Ycf1 in the assembly of ACCase has been proposed, based on this correlation (Bölter and Soll 2017). These three genes are notoriously variable and difficult to annotate, such that it is difficult to be sure whether a particular plastid sequence is nonfunctional or simply particularly divergent (Nakai 2015), and indeed we are not entirely certain of the functional status of *accD* in the Droseraceae. A concerted effort to reanalyze the annotations of these genes across land plant plastid genomes would be difficult, but worthwhile.

Besides the loss of “essential” protein-coding genes, the Droseraceae plastid genomes are also missing several tRNA genes found in almost all other plastid genomes. For example, *Dr.erythrorhiza* is missing two essential tRNA genes (*trnA-UGC* and *trnI-GAU*) and *D.muscipula* is missing *trnV-UAC*. Loss of plastid tRNA genes is common in parasitic plants that have lost the ability to photosynthesize (Wolfe, Morden, Ems, et al. 1992; Delannoy et al. 2011) and thus no longer require high rates of protein translation in their plastids but is unusual in photosynthetic plants. As plastid translation must occur in the Droseraceae, we presume that the missing tRNAs can be imported from the cytosol. All three of the equivalent tRNAs can be imported into plant mitochondria (Kumar et al. 1996), so it may simply be a question of co-opting the requisite machinery, but the process is likely to limit the maximal translation rates possible in Droseraceae plastids (Salinas et al. 2012). The rearrangement of the rRNA operons may also have implications for plastid translation. The typical *rrn16-trnI-trnA-rrn23-rrn4.5-rrn5* structure is highly conserved in plastids, and almost always within the inverted repeat, which helps preserve the sequence (Wolfe et al. 1987) and doubles the copy number of the *rrn* genes to permit high rates of ribosome synthesis. Within the Droseraceae, several alterations to this operon have occurred. Deletions between the *rrn16* and *rrn23* genes have removed part of *trnA* and *trnI* in *Dr.rotundifolia* and *Dr.erythrorhiza*, and severe contraction of the inverted repeats in *Dionaea* have left the genome with only one complete copy of the *rrn* operon, plus a second copy of *rrn16*. These rearrangements must have consequences for the processing of rRNA precursors and are likely to limit the maximal rate of plastid ribosome biogenesis.

#### Loss of *ndh* Genes in Carnivorous Plants

The genome rearrangements, gene losses, and general variability of the plastid genomes in the Droseraceae are reminiscent of what has been observed in other plants that are no longer entirely reliant on photosynthesis for energy and nutrients, such as hemiparasitic plants (McNeal, Kuehl, et al. 2007), partial mycoheterotrophs (e.g., orchids: Kim et al. 2015; Lin et al. 2017), and other carnivorous plants such as the Lentibulariaceae (Wicke et al. 2014). In particular, common to almost all of these plants is the early loss of the 11 *ndh* genes encoding subunits of the ferredoxin-plastoquinone oxidoreductase (NDH) complex (Ruhlman et al. 2015; Silva et al. 2016). This complex is related to mitochondrial complex I (NADH-ubiquinone oxidoreductase) and is thought to be able to transport protons across the thylakoid membrane in concert with cyclic electron flow around photosystem I, and in doing so can adjust the relative yield of ATP or reductant from photosynthetic electron flow (Shikanai 2016). The exact physiological role of the complex in most plants is still rather unclear, as mutants lacking it show few obvious abnormalities. It has variously been proposed to provide advantages under low light, high light, cold, or other “stress” conditions (Yamori et al. 2011). The loss of the NDH complex in partially parasitic, mycoheterotrophic, or carnivorous plants (and some other groups of plants with extensive mycorrhizal associations, such as the Pinaceae) may represent a loss of selection for optimal photosynthesis (Ruhlman et al. 2015), or it may be hinting at a specific function required only in fully autotrophic plants, presumably linked to nutrient assimilation. One candidate would be assimilation of nitrate, which requires photosynthesis-derived reductant.

## Conclusion

The gene loss patterns observed here are similar to those in carnivorous *Utricularia*, *Pinguicula*, and *Genlisea* (Wicke et al. 2014; Silva et al. 2016). Finding comparable patterns in the phylogenetically distinct Droseraceae strongly supports convergent evolution in the plastid genome, presumably driven by the switch to a carnivorous lifestyle. The changes observed here in plastid gene content also resemble those found in parasitic (e.g., McNeal, Arumugunathan, et al. 2007; McNeal, Kuehl, et al. 2007; McNeal et al. 2009) and mycoheterotrophic plants (Graham et al. 2017), supporting a broader hypothesis of convergent plastid genome alterations in plants where there is a switch from photoautotrophy to a partially or fully heterotrophic mode of nutrition.

## Supplementary Material

Supplementary data are available at *Genome Biology and Evolution* online.

## Supplementary Material

Supplementary Data

## Footnotes

**Data deposition:** The full sets of unassembled reads are available at SRA under the accessions SRR7072322–SRR7072325 and SRR7072766–SRR7072769. The assembled, annotated sequences are available at GenBank under the accessions KY651214 (*Drosera erythrorhiza*), KY679199 (*Drosera regia*), KY679200 (*Aldrovanda vesiculosa*), and KY679201 (*Dionaea muscipula*).
