# Supplementary material for: Plastome-Wide Rearrangements and Gene Losses in Carnivorous Droseraceae
Source: Genome Biol Evol. 2019 Jan 10;11(2):472–85. doi: 10.1093/gbe/evz005 (PMC6380313; doi:10.1093/gbe/evz005)
Supplement: Supplementary Data [file evz005_supp.zip › Figure S1.pdf]

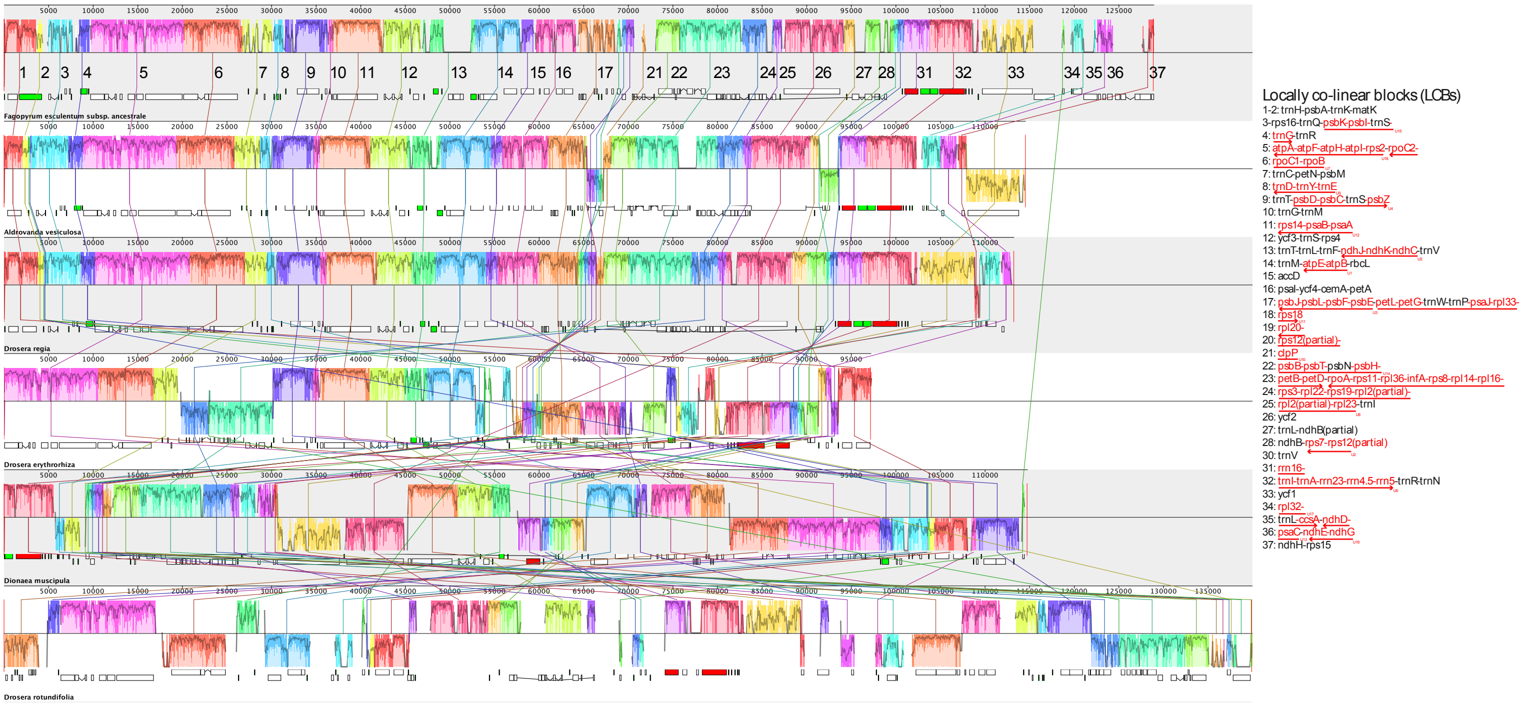

Supplementary Figure S1. Plastid genome rearrangements in the Droseraceae represented by locally co-linear blocks. The genome sequences were aligned with progressiveMauve v. 2015-02-25 (Darling et al. PLOS ONE 5: e11147, 2010) using default parameters. The 37 blocks identified by Mauve were annotated with the corresponding co-transcription units (those marked in red are conserved across most flowering plants and are listed in Table 2). Arrows indicate the direction of transcription of the units. The height of the similarity profile within the blocks corresponds to the average level of conservation in that region of the genome. Gene annotation is attached below each profile; CDS features are marked as white boxes, tRNAs as green boxes, and rRNAs as red boxes.
