# Supplementary material for: Plastome-Wide Rearrangements and Gene Losses in Carnivorous Droseraceae
Source: Genome Biol Evol. 2019 Jan 10;11(2):472–85. doi: 10.1093/gbe/evz005 (PMC6380313; doi:10.1093/gbe/evz005)
Supplement: Supplementary Data [file evz005_supp.zip › Figure S2.pdf]

a

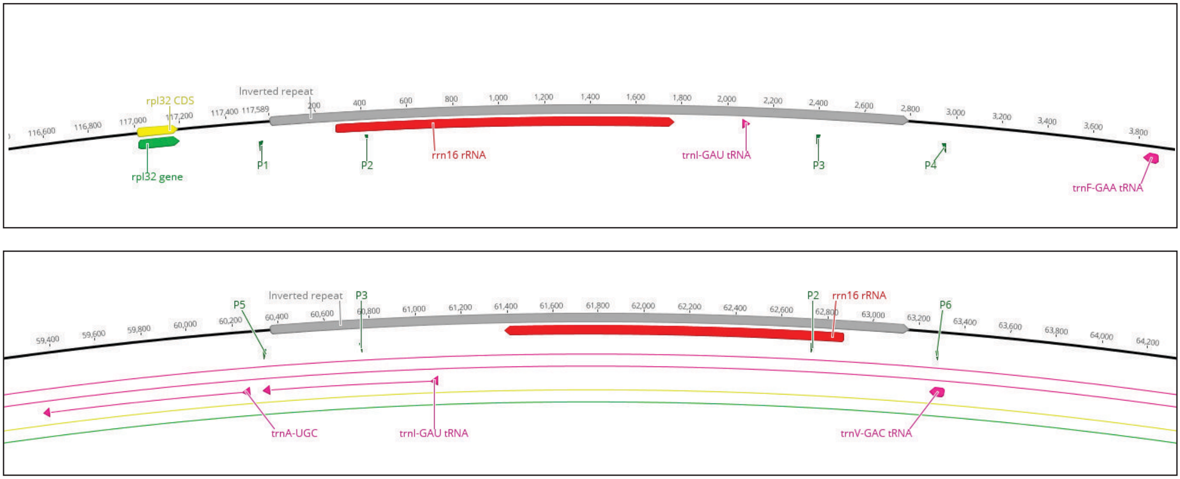

b

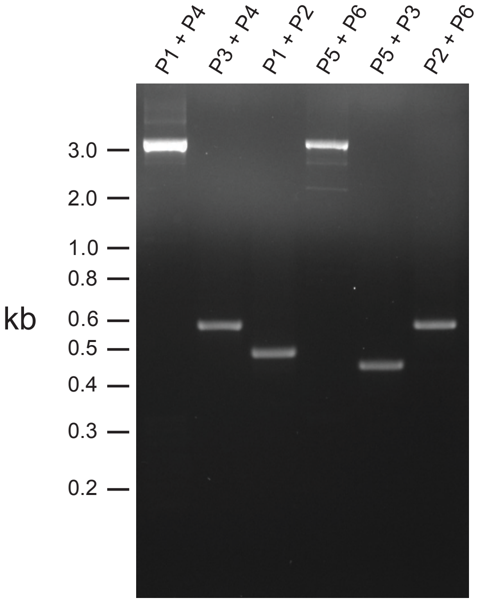

Supplementary Figure S2. Confirmation of the inverted repeat (IR) structure in the chloroplast genome of *Dionaea muscipula*. (a) Primers (P1-P6; Supplementary Table S1) were designed to amplify both versions of the inverted repeat determined by genome assembly, to confirm their structure and flanking regions. **(b)** PCR using the different primers shown in (A) confirmed that the IR structure determined by genome assembly was correct.
