# Supplementary material for: Plastome-Wide Rearrangements and Gene Losses in Carnivorous Droseraceae
Source: Genome Biol Evol. 2019 Jan 10;11(2):472–85. doi: 10.1093/gbe/evz005 (PMC6380313; doi:10.1093/gbe/evz005)
Supplement: Supplementary Data [file evz005_supp.zip › Supporting information legends.docx]

**Supporting information**

**Figure S1.** Confirmation of the inverted repeat (IR) structure in the chloroplast genome of *Dionaea muscipula*.

**Figure S2.** Alignment of ClpP1 sequences from a range of land plants including Droseraceae.

**Figure S3.** Plastid genome rearrangements in the Droseraceae represented by locally co-linear blocks.

**Table S1.** Primers used in this study.
