# Supplementary material for: Plastome-Wide Rearrangements and Gene Losses in Carnivorous Droseraceae
Source: Genome Biol Evol. 2019 Jan 10;11(2):472–85. doi: 10.1093/gbe/evz005 (PMC6380313; doi:10.1093/gbe/evz005)
Supplement: Supplementary Data [file evz005_supp.zip › Table S1.pdf]

Table S1. Primers used in this study.

Primer name	Sequence (5' to 3')
P1	GCGATAAAAAGAGGCTGGCG
P2	CCGTTTCCAGCTGTTGTTCC
P3	GCTATCTCTCGAGCACAGCG
P4	GCCTCCGGTTTTCTTCTTCG
P5	CTTGAACCAGAGACCTCACC
P6	GGAAGTCATCAGTTCGAGCC
